# Supplementary material for: Echinacea purpurea and Onopordum acanthium Combined Extracts Cause Immunomodulatory Effects in Lipopolysaccharide-Challenged Rats
Source: Plants (Basel). 2024 Dec 3;13(23):3397. doi: 10.3390/plants13233397 (PMC11644462; doi:10.3390/plants13233397)
Supplement: Supplementary file 1 [file plants-13-03397-s001.zip › plants-3330389-supplementary(1).pdf]

Echinacea purpurea and Onopordum acanthium Combined Extracts Cause Immunomodulatory Effect in Lipopolysaccha-ride-Challenged Rats

Supplementary material

Figure S1. Spectra of compounds of interest

1. Ferulic acid

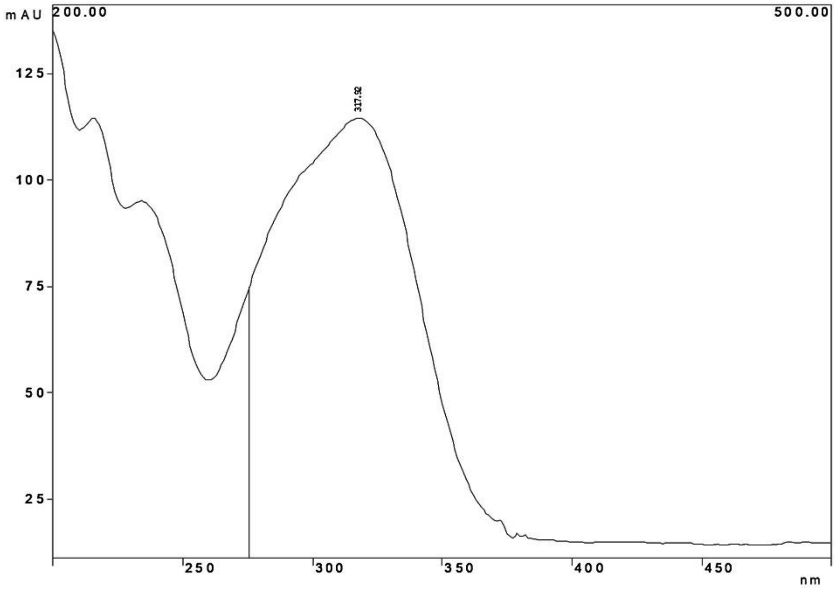

2. Caffeic acid

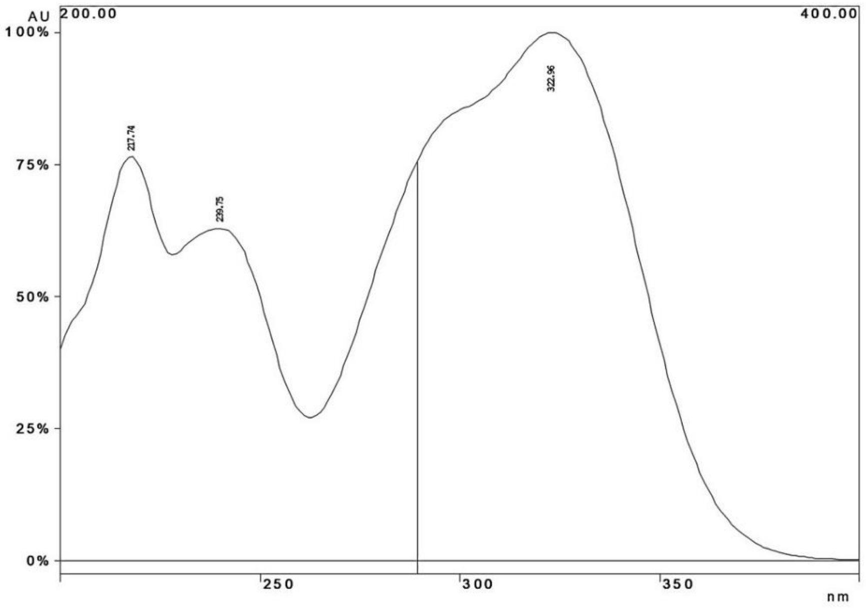

3. Caftaric acid

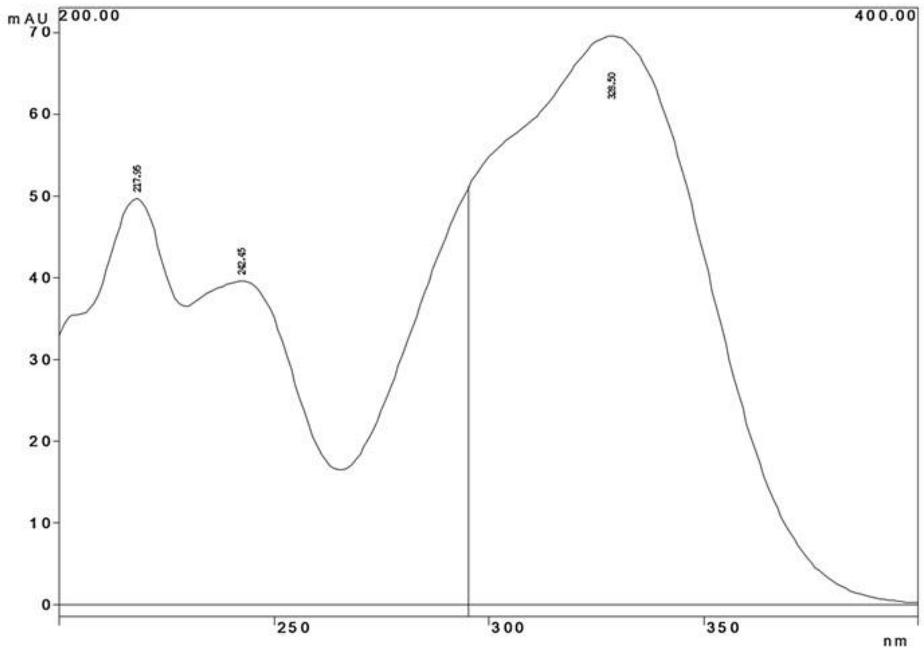

4. Chicoric acid

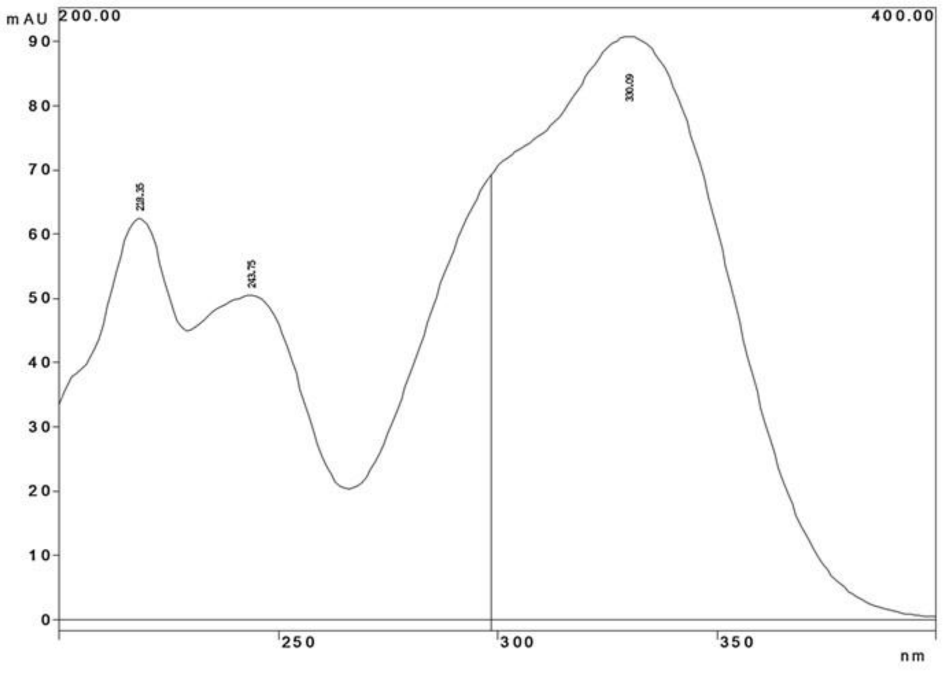

5. Cynarin

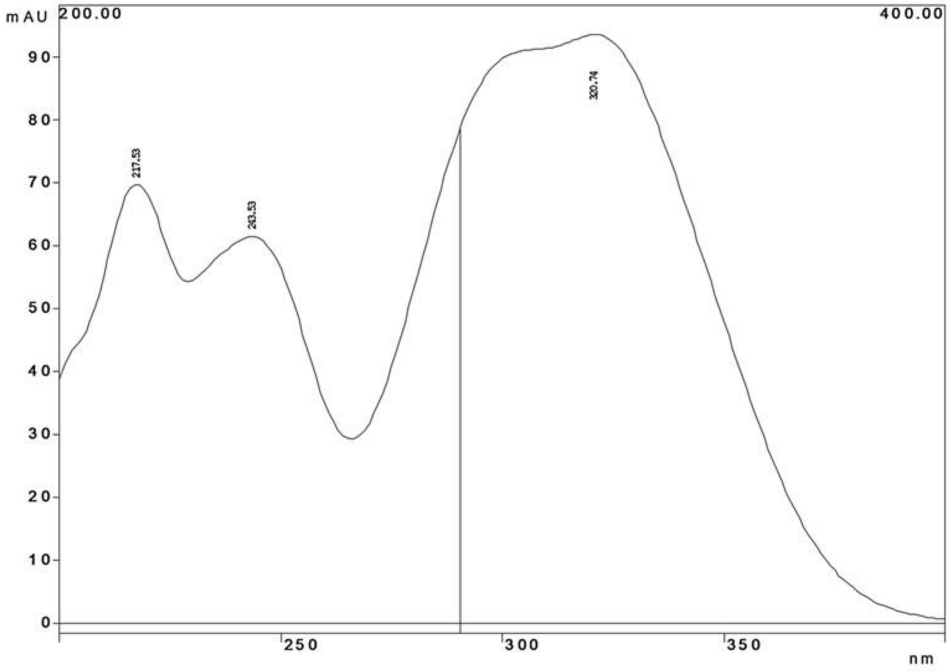

6. Echinacoside

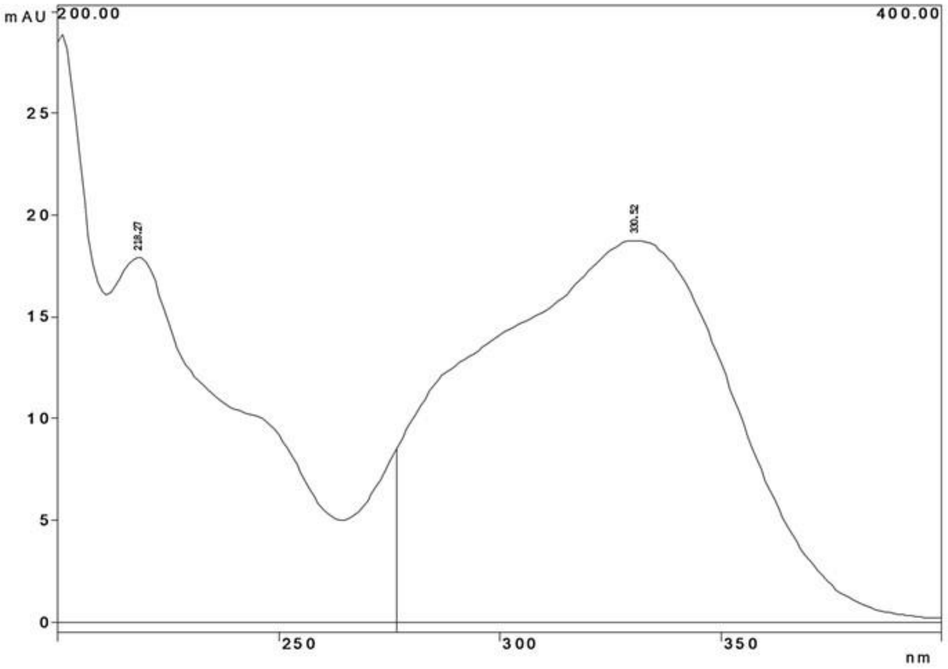

7. Chlorogenic acid

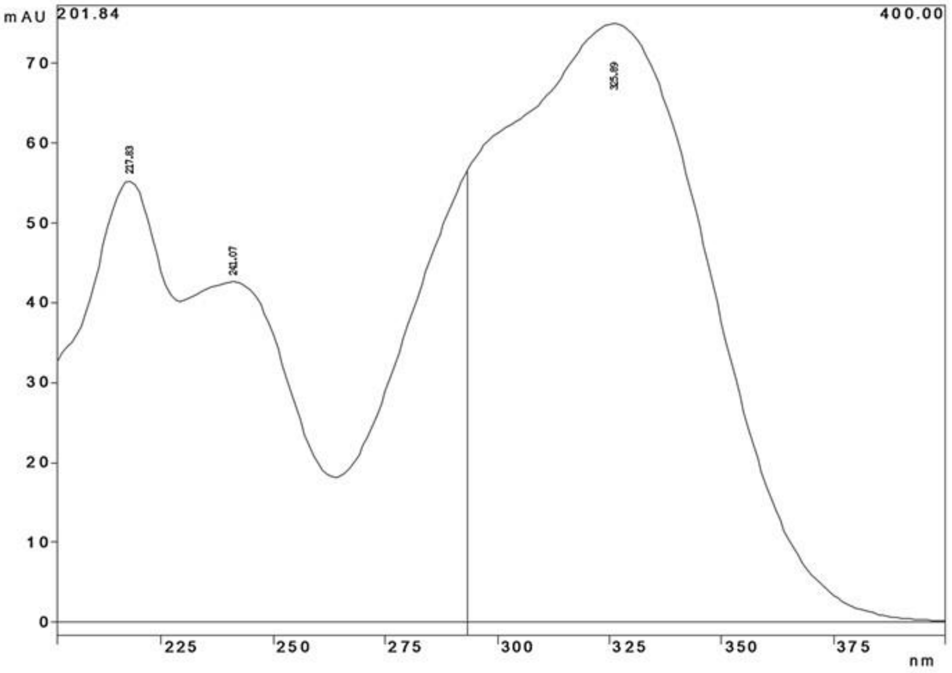

8. Quercetin

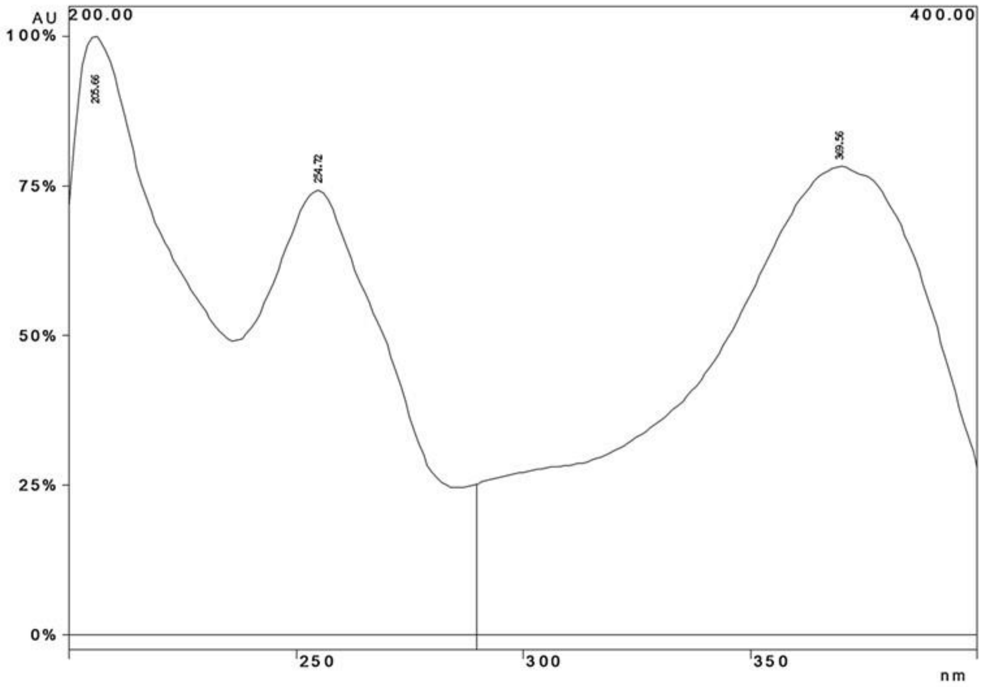

9. Apigenin

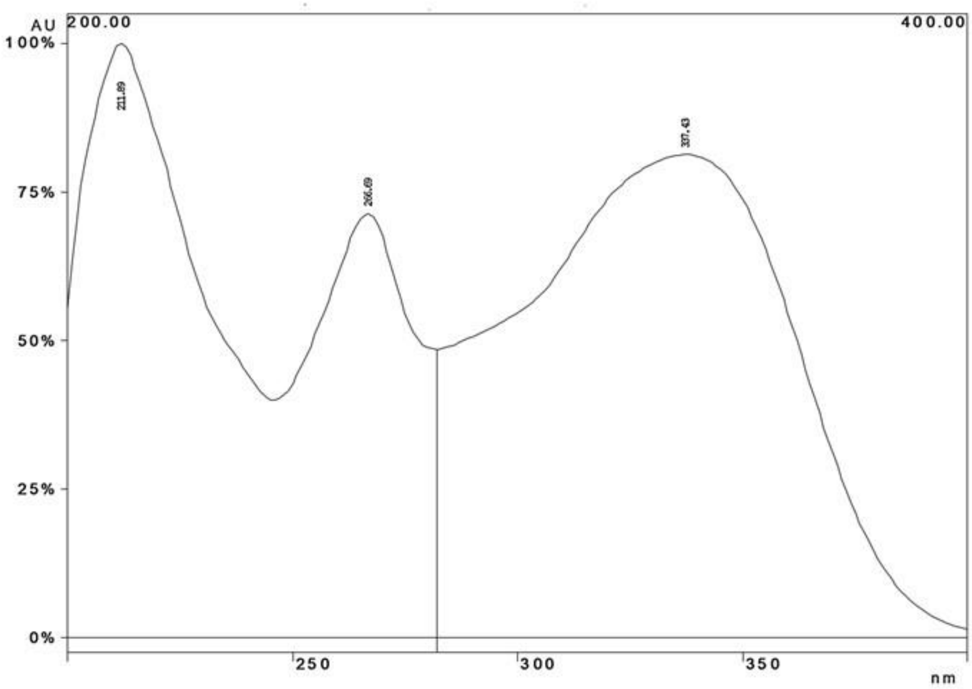

10. Rutin

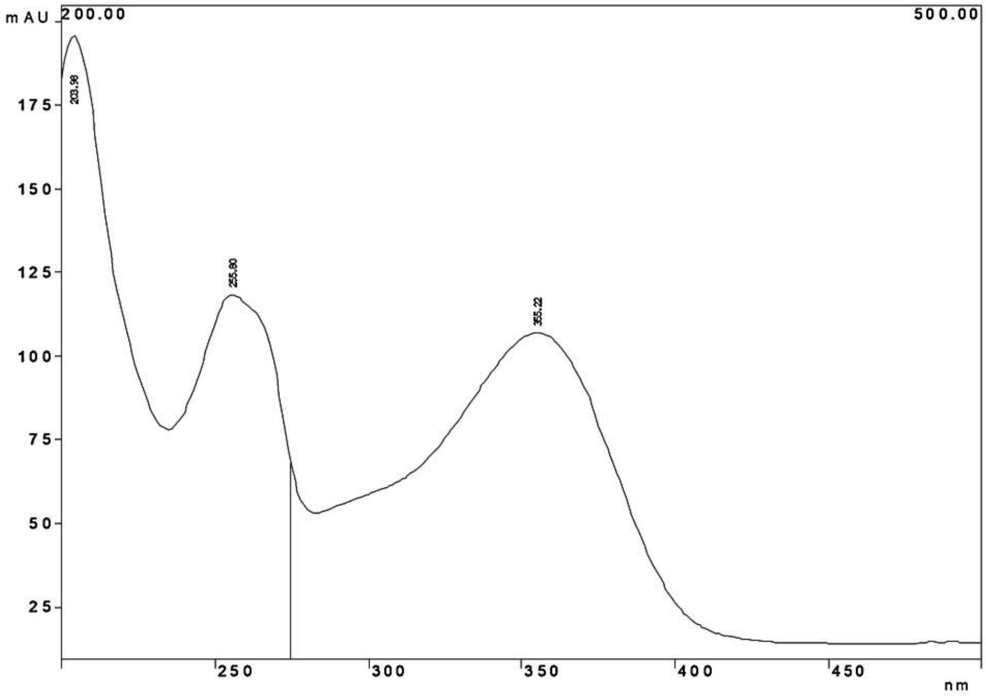

11. Scutellarin

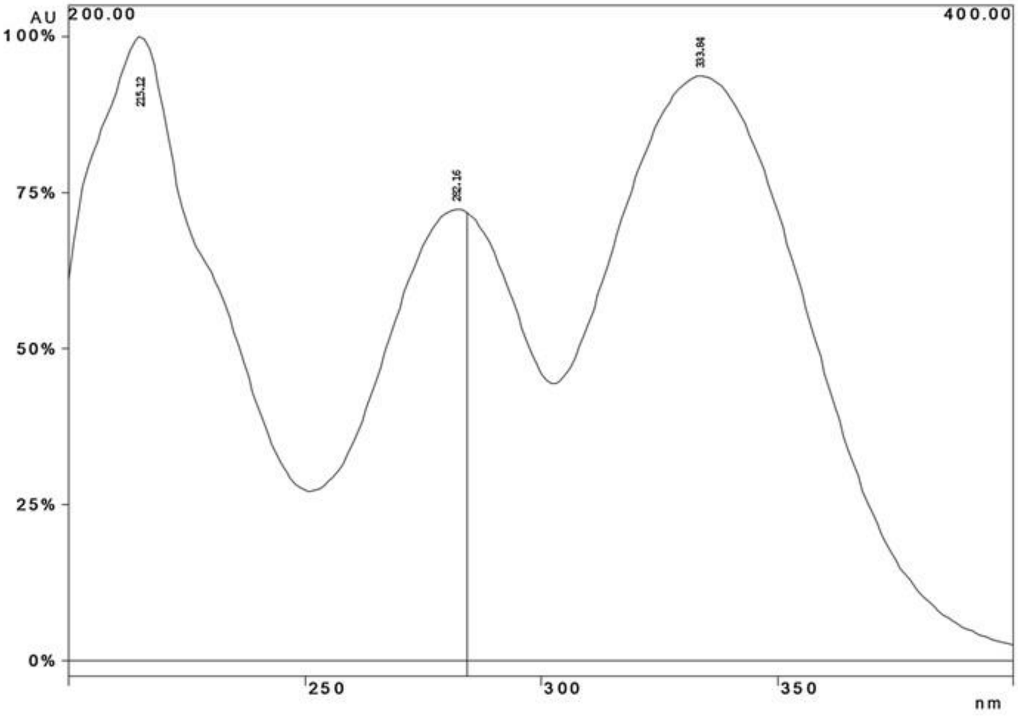

12. Arctigenin

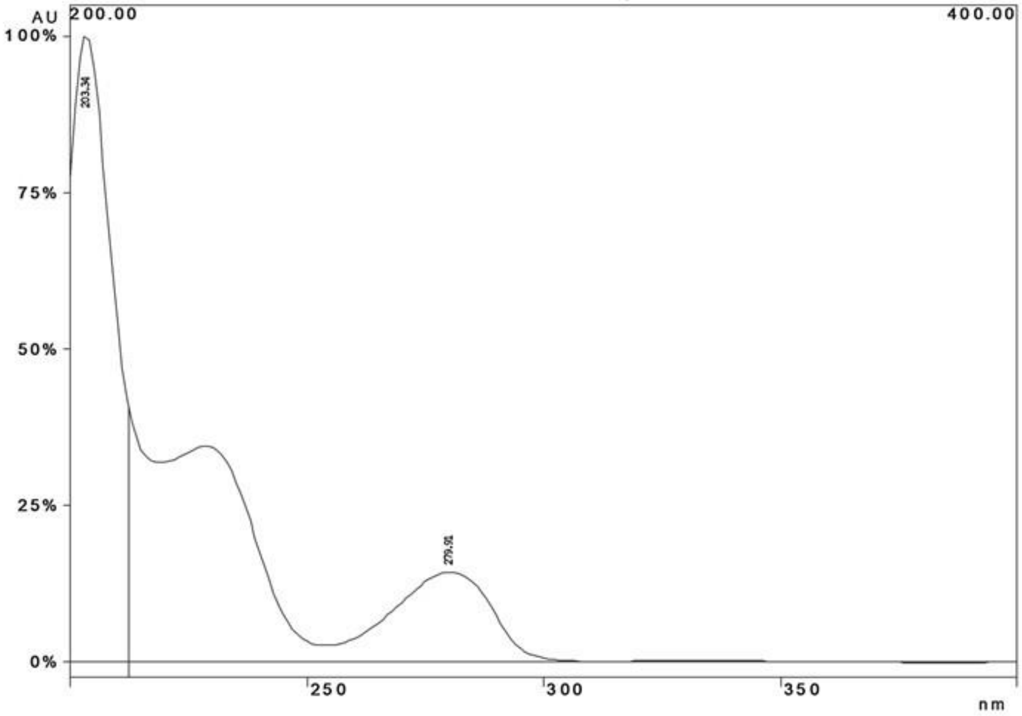

13. Myricetin

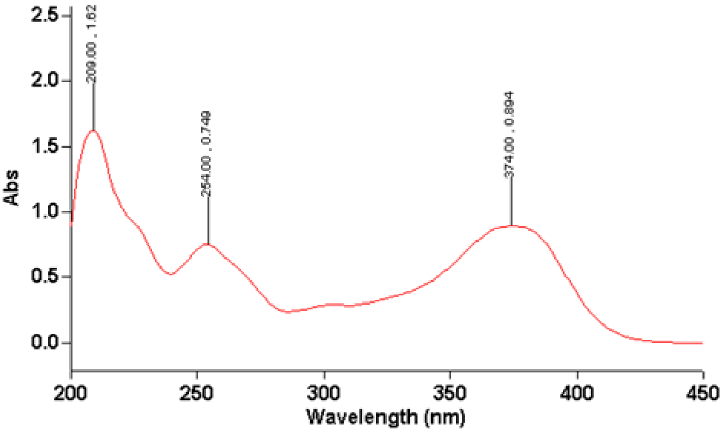

14. Epicatechin

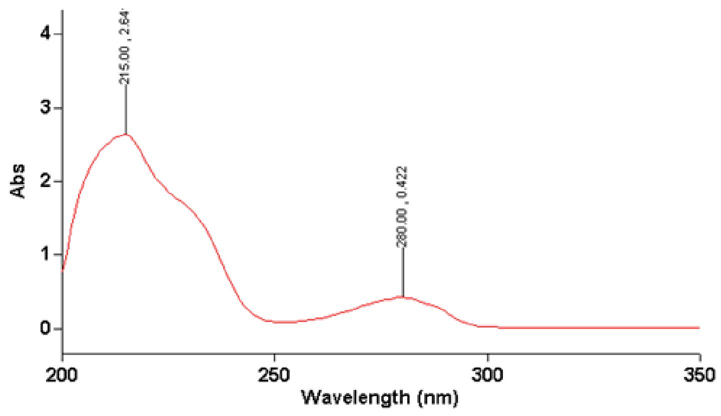

15. Neochlorogenic acid

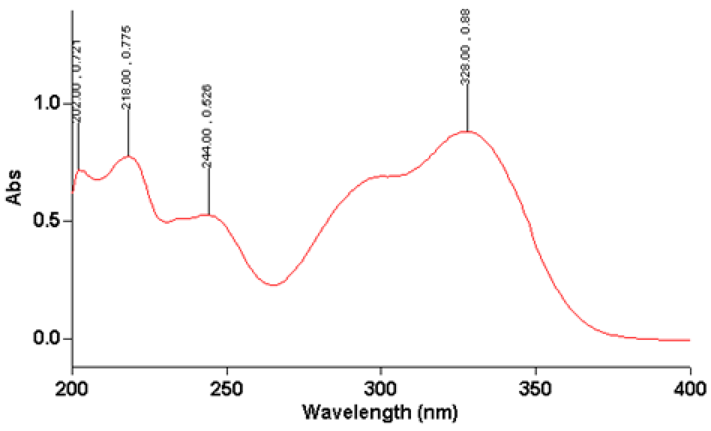

Figure S2. Chromatograms of extracts
Chromatogram of an individual extract of *E. purpurea* using the method described by Vlasheva et al. [29] (HPLC system – Varian)

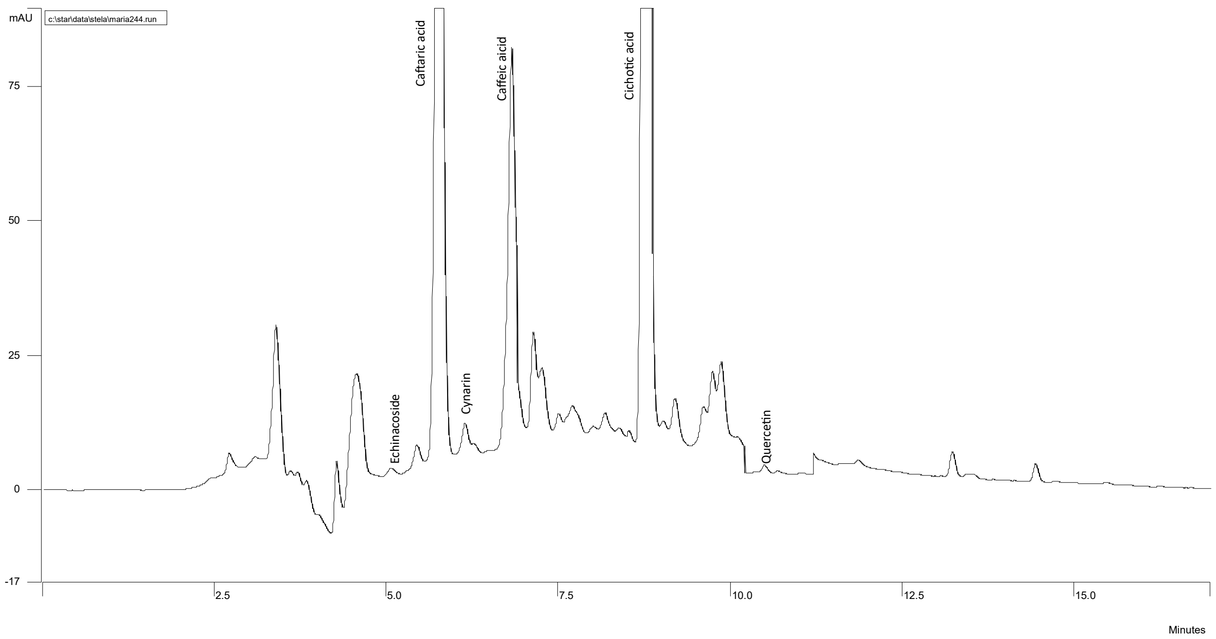

Chromatogram of Combination 1 using the method described by Vlasheva et al. [29] (HPLC system – Varian)

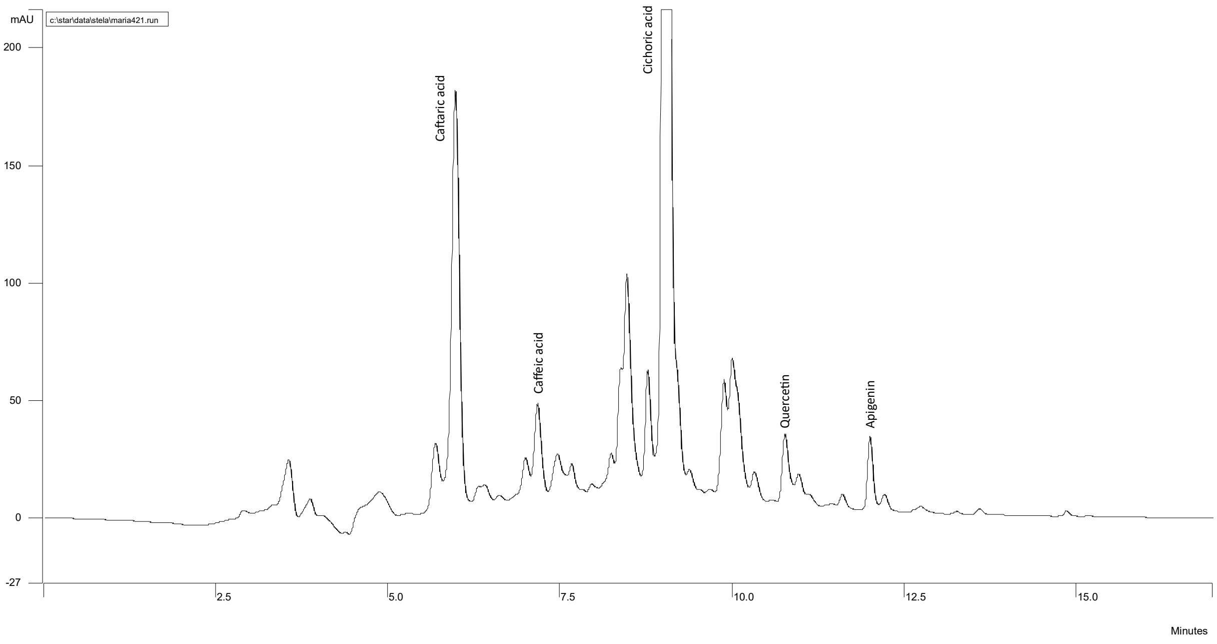

Chromatogram of Combination 2 using the method described by Vlasheva et al. [29] (HPLC system – Varian)

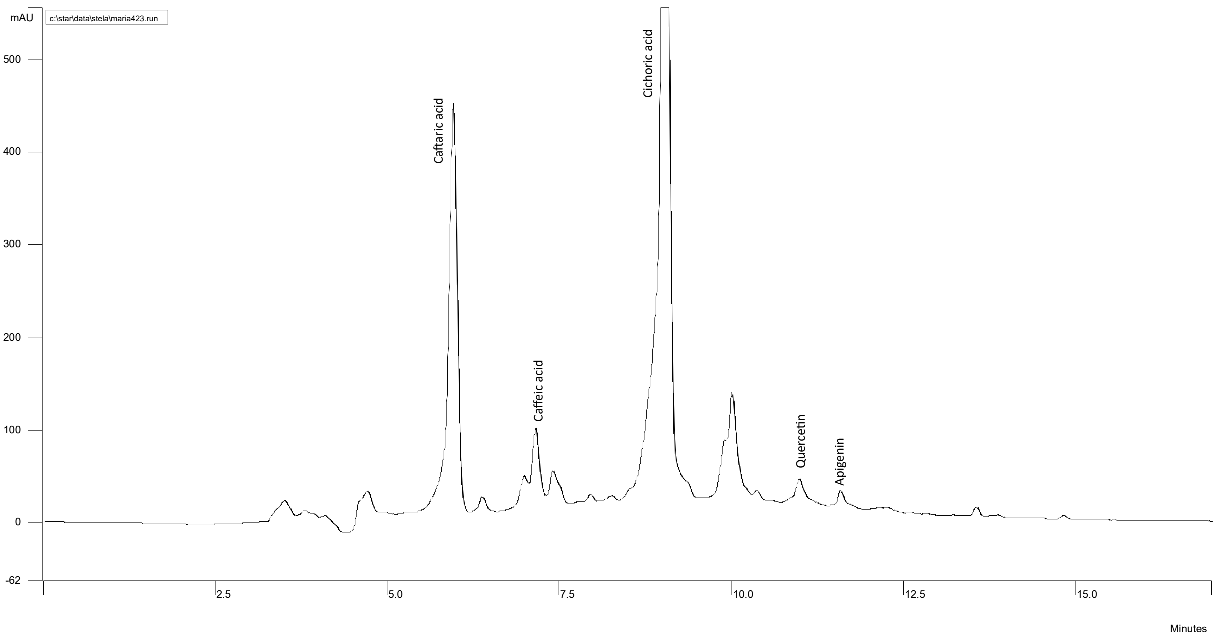

Chromatogram of individual extract of *O. acanthium* using a modified method (HPLC system – Varian)

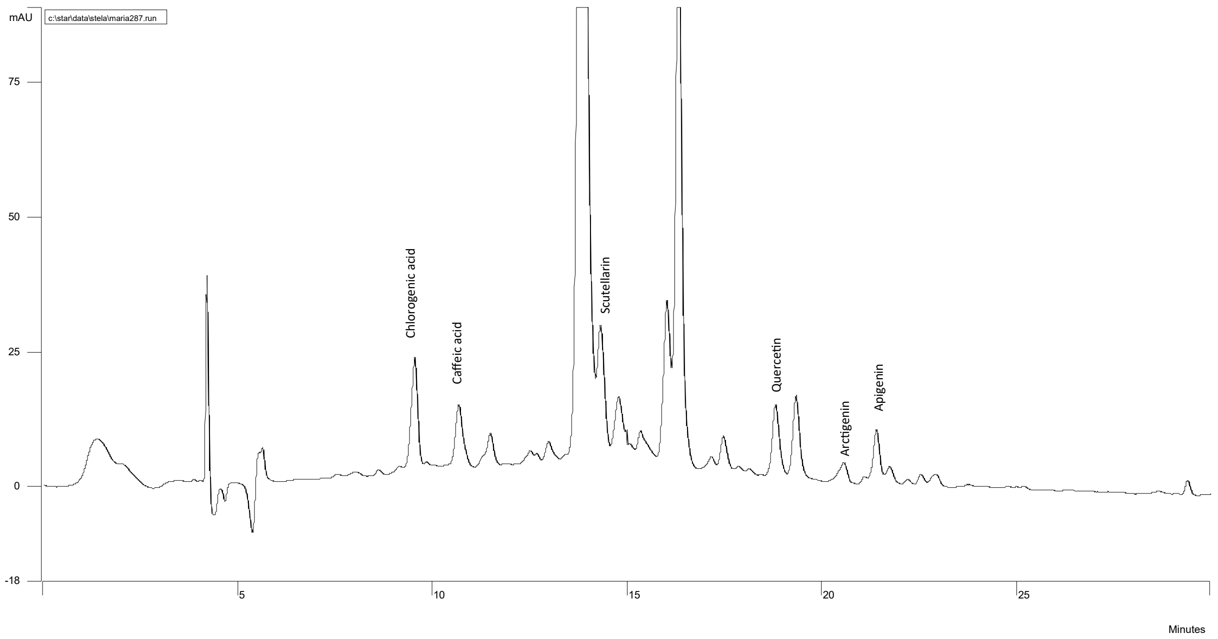

Chromatogram of Combination 1 using a modified method (HPLC system – Varian)

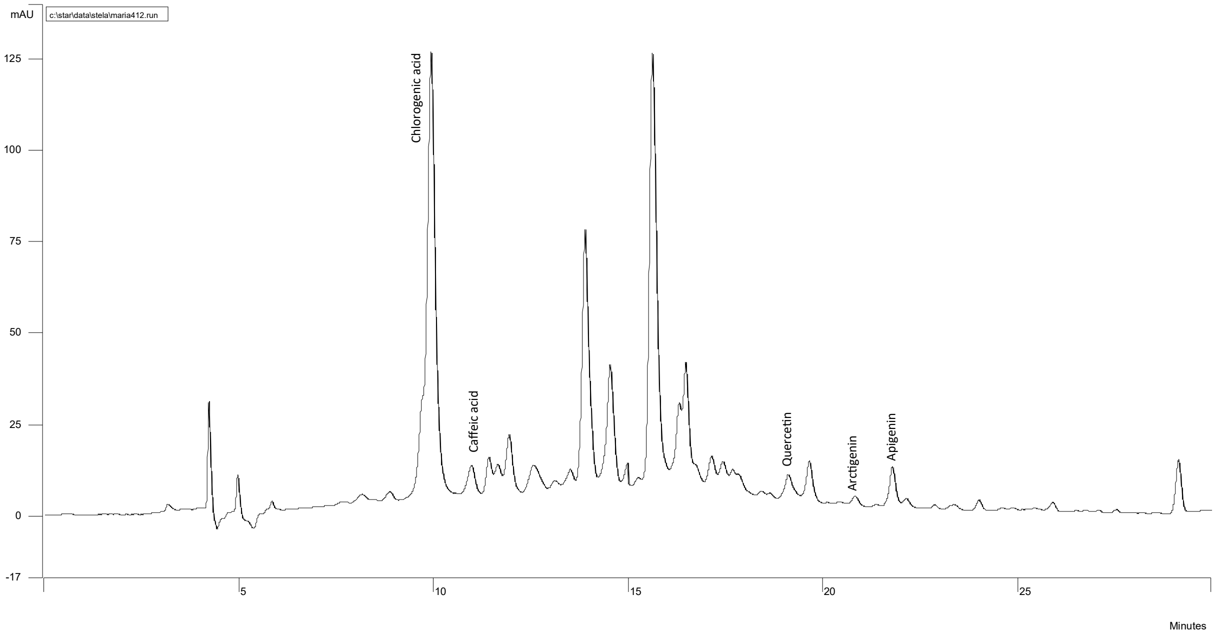

Chromatogram of Combination 2 using a modified method (HPLC system – Varian)

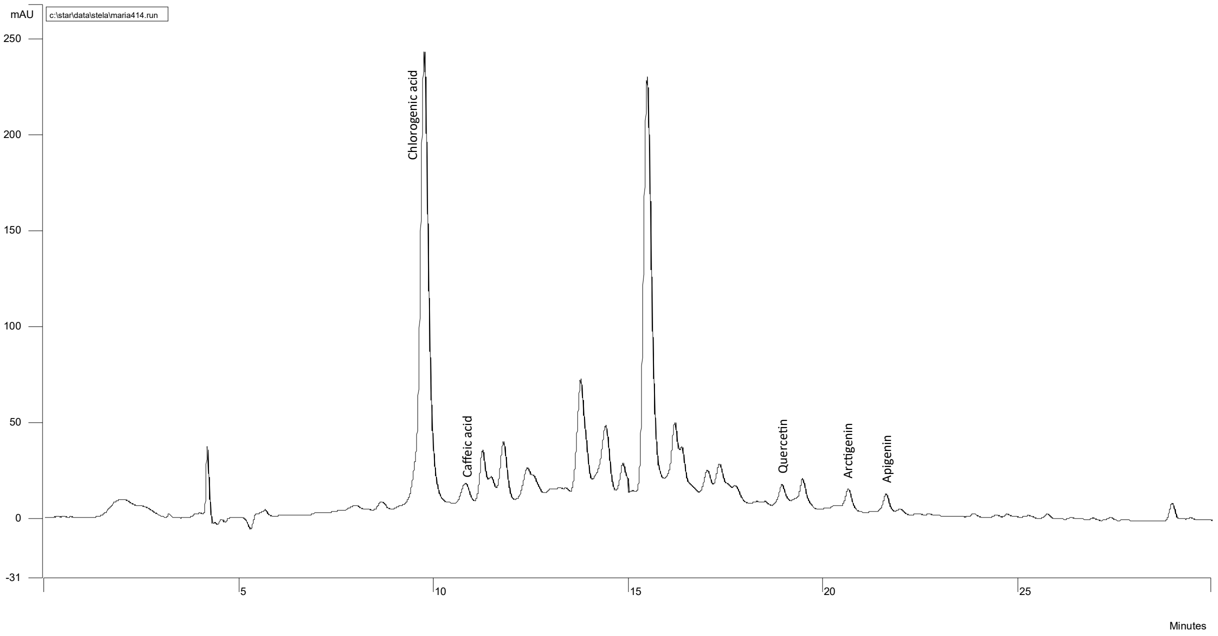

Chromatograms of *E. purpurea* and *O. acanthium* extracts using the method described by Teneva et al. [68] (UHPLC system – Shimadzu)

Echinacea purpurea

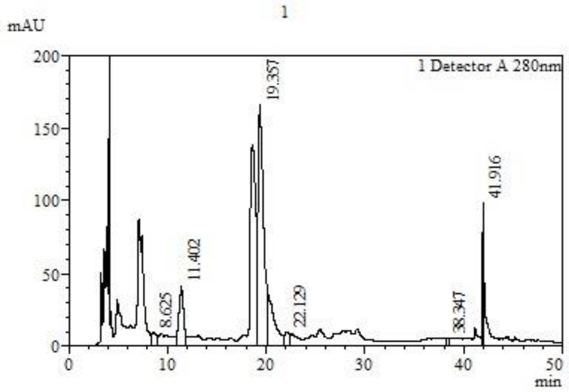

Onopordum acanthium

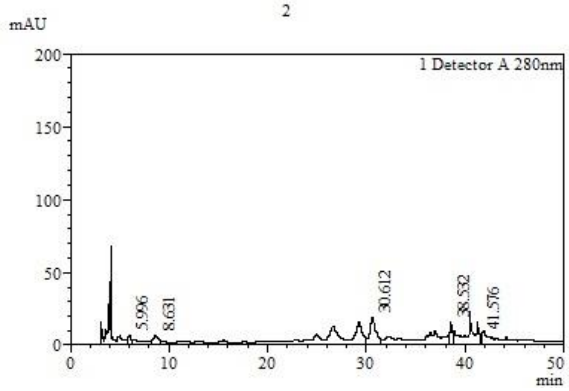

Compounds	RT (retention time), min
Neochlorogenic acid	5.9
Ferulic acid	19.3
Rutin	22.1
Myricetin	30.6
Epicatechin	41.6
